# Subjective signal strength distinguishes reality from imagination

**DOI:** 10.1038/s41467-023-37322-1

**Published:** 2023-03-23

**Authors:** Nadine Dijkstra, Stephen M. Fleming

**Affiliations:** 1 Wellcome Centre for Human Neuroimaging, University College London, London, UK; 2 Max Planck UCL Centre for Computational Psychiatry and Aging Research, University College London, London, UK; 3 Department of Experimental Psychology, University College London, London, UK

**Keywords:** Psychology, Cognitive neuroscience, Human behaviour

## Abstract

Humans are voracious imaginers, with internal simulations supporting memory, planning and decision-making. Because the neural mechanisms supporting imagery overlap with those supporting perception, a foundational question is how reality and imagination are kept apart. One possibility is that the intention to imagine is used to identify and discount self-generated signals during imagery. Alternatively, because internally generated signals are generally weaker, sensory strength is used to index reality. Traditional psychology experiments struggle to investigate this issue as subjects can rapidly learn that real stimuli are in play. Here, we combined one-trial-per-participant psychophysics with computational modelling and neuroimaging to show that imagined and perceived signals are in fact intermixed, with judgments of reality being determined by whether this intermixed signal is strong enough to cross a reality threshold. A consequence of this account is that when virtual or imagined signals are strong enough, they become subjectively indistinguishable from reality.

## Abstract (web summary)

How humans distinguish perception from mental imagery is not well understood. Here, the authors show that reality judgements are based on the intensity of a mixture of imagined and real signals.

## Introduction

In order to function in complex environments, humans have evolved to move beyond stimulus-triggered responses to guide behaviour via offline simulations, such as during navigation and planning^1^. Contemporary generative models of brain function propose that mental imagery relies on similar neural machinery to that engaged by veridical perception^2–4^, a hypothesis supported by neuroimaging data^5–8^. While allowing for a vast increase in cognitive sophistication, the existence of stimulus-independent processing poses a fundamental challenge for the nervous system: given that internally and externally triggered signals are often similar, there is considerable potential for confusing perception and imagery^9^. The capacity to resolve such confusion, and distinguish between imagination and reality, is known as perceptual reality monitoring.

One cue that the brain might use for reality monitoring is volition or intention. In the literature on sense of agency, the extent to which incoming sensory activity can be predicted from the intention to move is believed to generate the feeling that such activity is self-caused, rather than reflecting a change in the outside world^10,11^. Accordingly, sensory inputs congruent with self-triggered actions are attenuated because they are ‘explained away’ by a top-down intention signal. Hallucinations in disorders of reality monitoring such as schizophrenia are thus explained as reflecting disturbances in the forward model which predicts the sensory consequences of actions^12^. In this scenario, the sensory consequences of internally triggered speech or thoughts are not explained away and are therefore erroneously attributed to an external source^13–15^.

Anecdotal evidence that a similar mechanism might underlie perceptual reality monitoring in otherwise healthy observers comes from the Perky effect, which was demonstrated by Mary Cheves West Perky in 1910 and has achieved almost mythical status within imagery research^16,17^. In this study, participants were instructed to imagine various objects at a certain location on the wall while, unbeknownst to the participants, images of the same objects were simultaneously projected to the same location. All participants failed to notice the presence of these real stimuli, reflecting that “if I hadn’t known I was imagining, I would have thought it real” (Perky, 1910, p. 433).”

However, visual imagery is often also triggered automatically, outside of voluntary control and, despite the absence of a clear intention in these instances, such involuntary imagery is generally still not mistaken for reality^18,19^. Another cue that the brain might use to dissociate imagination and reality is sensory strength. The phenomenological experience of imagery tends to be much weaker and less clear than that of perception. This difference in sensory strength has recently been suggested to be a direct consequence of running the visual system backwards when engaged in mental imagery^20,21^. Due to the absence of bottom-up input during imagery, the excitatory neurons within the middle layer of primary visual cortex are not activated^22,23^, leading to lower activation levels overall. Furthermore, because imagery signals are likely to originate in higher-level areas which have larger receptive fields, the precision of sensory signals is also lower during imagery^24,25^. Accordingly, the sensory consequences of imagery might not need to be explained away because they are generally not strong enough to be mistaken for reality.

It has been surprisingly difficult to obtain empirical data with high enough statistical power that bears on these conjectures. Previous work has shown that imagery generally biases perception towards the imagined percept^26–28^ (but see^29^ for opposite effects) and that it can hamper or facilitate perceptual discrimination^30–33^. However, the consequence of these interactions for source judgements about whether signals reflect imagination or reality remain unclear. Moreover, in the context of a perceptual reality monitoring task, as soon as a participant realizes that an external stimulus might be presented, attention on subsequent trials will naturally be directed externally, biasing interpretation of sensory signals back towards reality^34,35^. This might explain the steady decline and eventual reversal in reports of the Perky effect over the years as the development of modern technology made multi-trial psychophysics the norm in psychology labs (Supplementary Fig. 1).

**Fig. 1:**
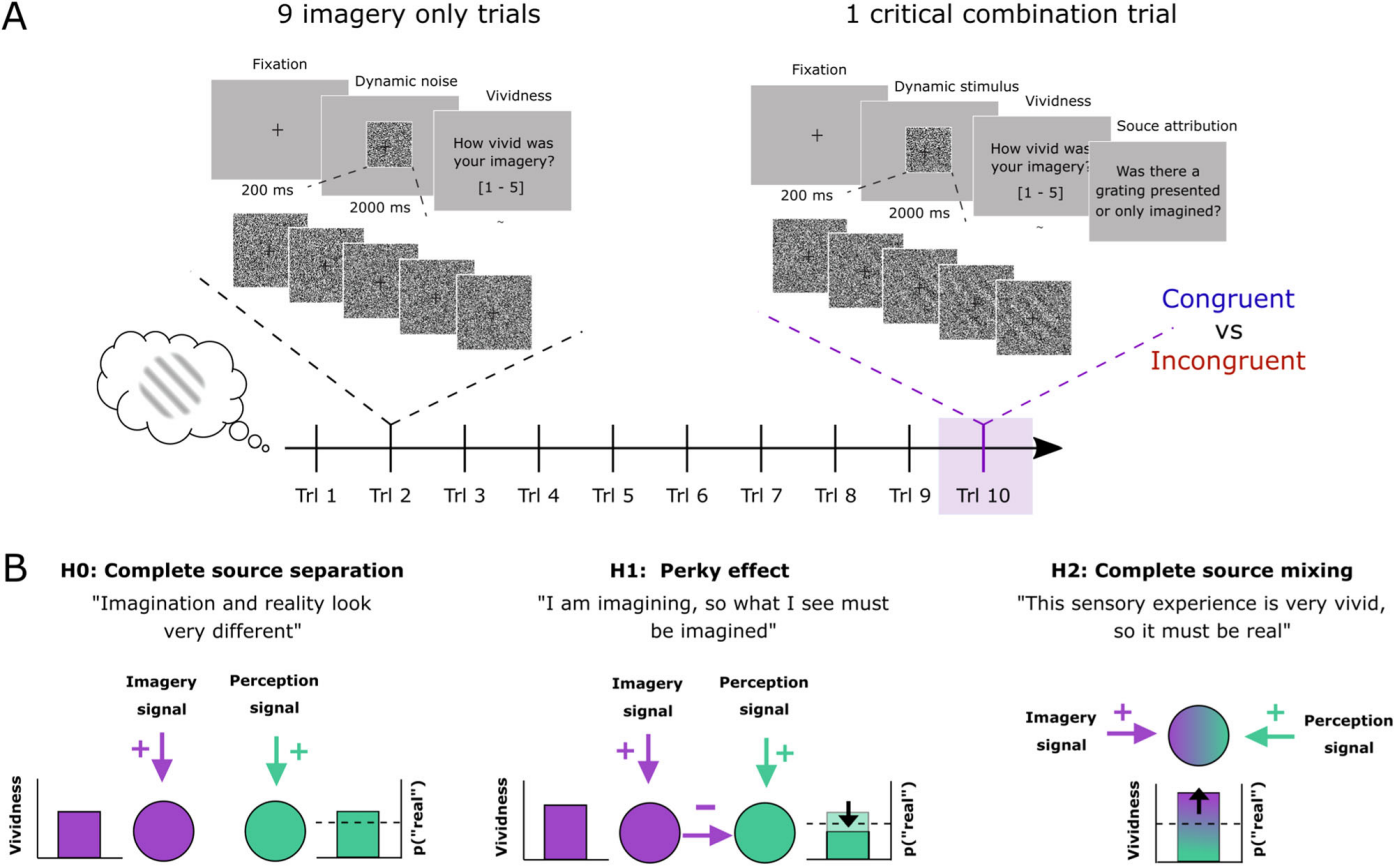
Experimental design and theoretical accounts of reality monitoring. **A** We employed online psychophysics to test reality monitoring in a statistically robust manner. Participants were instructed to imagine oriented gratings while looking at dynamic noise. On the final, critical trial, a grating with either the same (congruent) or perpendicular (incongruent) orientation to the imagined stimulus gradually became more visible until it was around detection threshold. Participants were then asked whether they thought a stimulus was presented on the last trial or if what they saw was imagined. Importantly, each participant only performed one critical, reality monitoring trial ensuring they remained naïve about the potential presence of external stimuli. **B** We compared three theoretical accounts of perceptual reality monitoring: H0 source separation, H1 Perky effect^11^ and H2 complete source mixing.

In the current study we combine computational modelling with recent developments in large-scale online data collection^35,36^ and single-trial psychophysics to test different models of perceptual reality monitoring in a statistically robust fashion. We validate model predictions about underlying brain mechanisms using multivariate pattern analysis of neuroimaging data. In a behavioural study, participants were instructed to vividly imagine a stimulus while looking at dynamic noise and then report the vividness of their imagery (Fig. 1A). Then, unbeknownst to the participant, similar to Perky’s (1910) study, on a final, critical trial, a stimulus that was either congruent or incongruent with participants’ imagery gradually appeared in the noise until it was around detection threshold. After again reporting their imagery vividness on this critical trial, participants were asked whether they thought a real stimulus had been presented on the last trial, or whether what they had experienced was only imagined. Participants’ answers to this last question provided a direct, unbiased test of perceptual reality monitoring, in the absence of expectations or prior instructions that external stimuli might be presented.

## Results

### Competing models for perceptual reality monitoring

We combined these large-scale single-trial psychophysical data with computational modelling and neuroimaging to distinguish between three hypothesized mechanisms for perceptual reality monitoring. According to our null model, the subjective experiences of imagery and perception are distinct and they do not get confused (Fig. 1B; H0). In this scenario, imagery vividness and perceptual visibility (an inference as to whether a real stimulus is present) are each determined by distinct computations. This model suggests that imagery and perception in fact rely on distinct neural populations (e.g. in different cortical layers^22,23^) but that the overlap found in previous studies might for example be an artefact of the poor resolution of neuroimaging methods which look at the average activation over many thousands of neurons^37^. Accordingly, this null model (H0) predicts that whether imagery is congruent or not with perception is immaterial for reality judgements, and that reality judgements do not influence reported imagery vividness.

Our second hypothesis (H1) is inspired by Perky’s findings and captures the idea that sensory signals in line with an imagery intention are inferred to reflect imagination. It predicts that if the same content is both imagined and perceived, external input is ‘explained away’ as reflecting imagery (Fig. 1B; H1). This can be modelled as a suppressive influence of imagery on perception, decreasing the probability that a stimulus is judged to be real when that stimulus is also imagined. Accordingly, when a congruent stimulus is judged to be imagined this means that the suppressive influence of imagery was strong enough to suppress the sensory input. Therefore, congruent imagery judgements should be associated with higher imagery vividness reports than congruent reality judgements.

Finally, if imagery and perception are supported by similar computations and imagery signals are not explained away, we might expect the subjective experiences of imagery and perception to only be distinguishable based on their relative strength (Fig. 1B; H2). In this scenario, imagining and perceiving the same content would lead to an increase in both imagery vividness as well as perceptual visibility, because both signals are added together to create one, inseparable sensory experience. Reality monitoring is then implemented by judging whether this subjective mixture of perception and imagery crosses a reality threshold (Fig. 1B; H2). Previous studies reporting more liberal perceptual detection criteria during simultaneous congruent imagery would be in line with this model^35,38^. Furthermore, because imagery and perception are intermixed and reality judgements are based on the sensory strength of the mixed signal, this model also predicts that when stimuli are judged to reflect reality, they should be associated with stronger ratings of imagery vividness compared to when they are judged to reflect imagination.

**Fig. 2:**
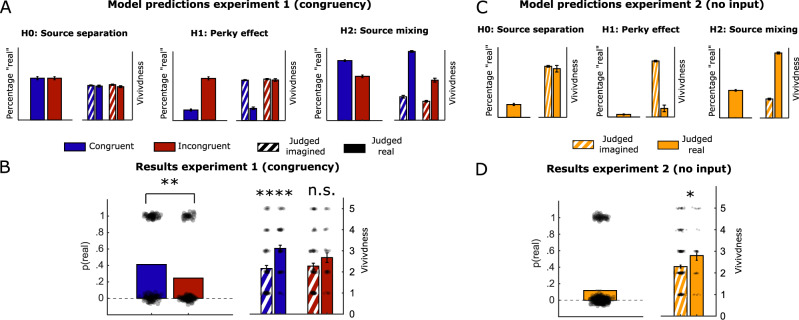
Model predictions and results. **A** Simulations of the three alternative theoretical models. Predicted percentage of trials judged as “real” (left hand bars) and imagery vividness (right hand bars) separated by condition (congruent, incongruent) and, for vividness ratings, reality judgment. Error bars indicate the standard error of the mean (SEM) over simulation samples. For H0: *n* = 1898; H1: *n* = 1872; H2: *n* = 1682 independent simulation samples. **B** Empirical data for reality judgments and vividness from Experiment 1. Data are presented as mean values +/− SEM and dots indicate individual participants, *n* = 274 independent participants. A logistic regression indicated a main effect of condition on reality judgements (*β*(1) = 0.860 (CI = 0.31–1.41), *p* = 0.002 (uncorrected), *OR* = 0.423). A multinomial regression indicated that there was a significant difference in vividness for real versus imagined judgements for the congruent condition (*β(1)*=0.40 (*CI* = 0.22– 0.58) *p* = 0.000013 (uncorrected)) but not for the incongruent condition (*β*(1)=0.19 (*CI* = −0.03–0.40), *p* = 0.092 (uncorrected)) **C** Model simulations as in (**A**), but now for the case with no external input. Data are presented as mean values + /− SEM over simulation samples, for H0: *n* = 902; H1: *n* = 879; H2: *n* = 929 independent simulation samples **D**. Empirical findings for Experiment 2. A multinomial regression indicated a significance difference in vividness for real versus imagined judgements (*β*(1)=0.22, *p* = 0.013, *CI* = 0.05–0.40). Data are presented as mean values +/− SEM over participants, *n* = 339 independent participants. ^*^p < 0.05; ^**^p < 0.005; ^***^p < 0.0005; ^****^p < 0.00005 all two-sided. Source data are provided as a Source Data file.

We simulated the predicted pattern of results in the two conditions for each of these three models within a signal detection theory framework^39^ (see Methods for details). Critically, as shown in Fig. 2A, each hypothesis makes qualitatively different predictions for the patterns of imagery vividness and perceptual reality judgments on the final, critical trial across the conditions in our experiment. Complete source separation (H0) predicts no difference between congruent and incongruent imagery conditions in terms of experienced vividness or the likelihood an external stimulus is judged as real (Fig. 2A, left). This is because under this model imagery and perception are always distinguishable, even if they overlap in content (the congruent condition). In contrast, the Perky effect (H1) predicts that congruent perceptual signals are incorporated into the imagery experience such that vivid, congruent imagery leads to a *decrease* in the likelihood a stimulus is judged as real (Fig. 2A, middle). Finally, complete source mixing (H2) predicts that internal and external signals are intermixed and this combined signal underpins judgments of both stimulus presence and imagery vividness. This account predicts bidirectional influences between detection and vividness on congruent imagery trials, such that there is both a higher probability of judging a stimulus as real (relative to incongruent imagery), and, counter-intuitively, when a stimulus is judged to be real, the vividness of imagery should also be higher (Fig. 2A, right). We also explored a variation of the source mixing model that contained an extra parameter which allowed for the weighting of internal and external signals to be different for source judgements and imagery judgements (Supplementary Fig. 3A). This model predicted qualitatively similar results as the more parsimonious source mixing model (Supplementary Fig. 3B), and therefore in what follows we focus on the simpler one-parameter model.

### Perception and imagery are subjectively intermixed

The results of Experiment 1 (*N* = 272) are shown in Fig. 2B. We found that the probability of judging a stimulus to be real was higher in the congruent condition (0.41) compared to the incongruent condition (0.25; OR = 0.423, *β* = 0.860, SE = 0.279, *p* = 0.002, CI = 0.31–1.41; Fig. 2B, left) and that during the congruent condition, participants who judged the critical trial to be real also indicated higher imagery vividness for that trial (*M* = 3.12, *SD* = 1.17) compared to participants who judged the critical trial to be imagined (*M* = 2.16, *SD* = 1.32; *β(1)=0.40*, *p* = 0.000013, CI = 0.22–0.58). The difference in vividness in the incongruent condition was not significant (*β(1)=0.40*, *p* = 0.092, *CI* = −0.03 – 0.40). This qualitative pattern of results is exactly as predicted under the complete source mixing hypothesis (Fig. 2A; right), and goes against the other two hypotheses which would either predict no differences in reality judgments and vividness reports (H0), or the opposite direction of effect (H1). Note that the source mixing model also predicts a difference in vividness between real and imagined judgements in the incongruent condition and while this is numerically indeed the case, this difference is not significant, likely due to the increased noise level in our data compared to the simulations. Together, this combination of simple models with large-scale one-shot psychophysics reveals a failure of perceptual reality monitoring – when a stimulus is judged as real, participants experience greater imagery vividness, and in turn congruent imagery leads to a greater likelihood of a stimulus being judged as real.

One potential concern is that due to the online nature of these experiments, participants were less engaged with either imagination, perception, or both. To investigate this possibility, we simulated how changes in task engagement influenced the predicted patterns of results for each of our models. We modelled task engagement as influencing either external attention (the scaling of sensory input), the strength of the imagery signal, or both. While task engagement did influence the overall predicted proportion of presence responses and vividness ratings in all models, the patterns of predicted condition differences remained unchanged when comparing between models (Supplementary Fig. 2). For example, the Perky model always predicted more congruent than incongruent misses while the intermixing model always predicted the reverse. This suggests that the qualitative differences between conditions we observe here cannot be explained based on an overall lack of task engagement. We also considered whether our results could be explained by individual differences in perceptual sensitivity between conditions, irrespective of imagery. In general, participants with a higher discrimination sensitivity d’ were more likely to respond that they saw a real stimulus (*F*(1) = 15.93, *p* < 0.001, *β* = 0.84*, CI:* 0.39–1.30). Critically, however, there was no difference in d’ between the conditions (*F*(1) = 2.851, *p* = 0.092, CI = −0.33– 0.026) nor an interaction between condition and reality judgement (*F*(1) = .753, *p* = 0.386), indicating that our effect also cannot be explained by individual differences in discrimination sensitivity.

An alternative explanation of our results is that participants may have been confused about the task instructions, and mistakenly reported the vividness of the stimulus on the critical trial rather than their imagery. According to this explanation, imagery and perception are in fact subjectively dissociable, but participants confused them when giving their ratings – perhaps due to the surprising appearance of an external stimulus on the final, critical trial. In order to rule out this possibility, we ran another experiment (Experiment 2, *N* = 339) in which we never presented a stimulus on the critical trial, meaning that participants could not mistakenly interpret the vividness of an external stimulus as imagery. The instructions were identical to Experiment 1. Importantly, the different models again make qualitatively distinct predictions about how vividness covaries with reality judgements on the critical trial – even in the absence of external input (see Fig. 2C). We found that the vividness of critical trials mistakenly judged to be real was higher (*M* = 2.8, *SD* = 1.36) compared to critical trials correctly judged to be imagined (*M* = 2.29, *SD* = 1.2; *β*(1) = 0.22, *p* = 0.013, *CI* = 0.05–0.40; Fig. 2D) – again in line with the source mixing hypothesis and qualitatively at odds with the predictions of the other two accounts.

### Neural correlates of sensory strength

Together, the results from Experiments 1 and 2 reveal that the overlap between imagery and perception places fundamental constraints on perceptual reality monitoring. A simple signal detection model accounted for these findings by hypothesising that the vividness of imagery and the visibility of perception rely on common latent variables (Fig. 1B; H2). If this is the case, we would expect similar neural substrates to track both the vividness of internally triggered images as well as the visibility of externally presented stimuli. We tested this prediction by reanalysing functional magnetic resonance imaging (fMRI) data collected while participants gave vividness and visibility ratings during imagery and perception, respectively^40^. In both tasks participants performed a forced-choice animacy discrimination task and rated the visibility/vividness of their visual experience on a 4-point scale (response mappings were randomized over trials to exclude motor contributions to vividness/visibility-related activations). During perception, the stimuli were presented very briefly followed by a backward mask, leading to variation in visibility. The imagery task was a retro-cue task in which participants imagined one of two previously perceived stimuli, without any physical stimulus being present, and without any detection judgment being required.

**Fig. 3:**
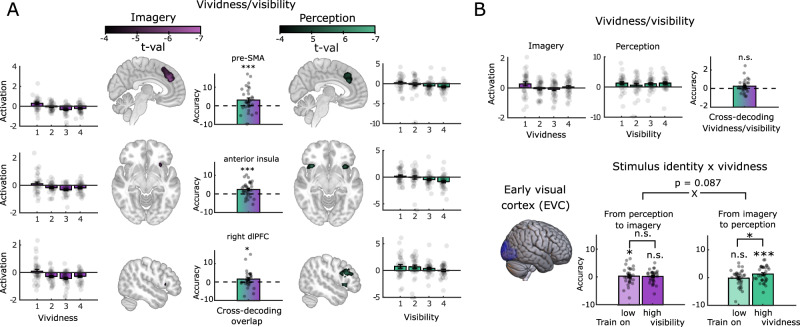
Neural representations of imagery vividness and perceptual visibility. **A** The outer panels show brain areas significantly modulated by imagery vividness (left) and perceptual visibility (right). The bars indicate the average activation per vividness/visibility level where 1 is low vividness/visibility and 4 is high vividness/visibility. The middle panel shows cross-decoding accuracy minus chance level (50%) between imagery vividness and perceptual visibility in the three brain areas that showed significant univariate modulation, indicating that not only are the same areas involved in tracking vividness and visibility, they also do so in a similar manner. Pre-SMA: pre-supplementary motor area, dlPFC: dorsolateral prefrontal cortex. *n* = 35 independent participants. Plotted brain regions showed a significant effect of vividness/visibility in a general linear model with whole-brain FDR correction applied at a *q*-value of 0.01 (two-sided) with all t(34)<−4.14. Significance of decoding accuracy was determined based on a one-sided permutation test within individual ROIs^84^; pre-SMA: *t*(34)=2.96, *p* = 0.0003, *d* = 0.5, *CI* = 0.51–0.55; anterior insula: *t*(34) = 2.96, *p* = 0.0008, *d* = 0.5, *CI* = 0.51–0.54; right dlPFC: *t*(34) =  2.37, *p* = 0.0097, *d* = 0.4, *CI* = 0.502–0.53, all uncorrected. **B** The influence of vividness/visibility on early visual cortex activity (EVC). The bottom left plot represents the location of the EVC region of interest (ROI) in blue. The top panel indicate the univariate effect of vividness *F*(26, 3) = 1.76, *p* = 0.18 (two-sided, uncorrected)/visibility (*F*(26, 3) = 0.35, *p* = 0.78 (two-sided, uncorrected) on overall activation level within the ROI (lefthand two plots) as well as on the cross-decoding accuracy (*t*(34) = 1.18, *p* = 0.13 (one-sided, uncorrected), *CI* = 0.5–0.52) between vividness and visibility (right plot). The panels on the bottom right indicate the influence of vividness/visibility on the overlap in representations of stimulus content between imagery and perception. Purple bars reflect training on perception and decoding during imagery and green bars reflect training on imagery and decoding during perception. Light bars reflect training on low visibility/vividness trials, and darker bars reflects training on high visibility/vividness trials. These results indicate that representations of stimulus content during imagery are more akin to those evoked on low-visibility perception trials, whereas representations of stimulus content during perception are more akin to those evoked during high vividness imagery. *n* = 33 independent participants. Errors bars represent SEM over participants, and dots represent individual participants; train on low visibility: *t*(32) =  0.77, *p* = 0.025 (one-sided, uncorrected), *CI* = 0.5–0.514; train on high visibility: *t*(32) = 0.78, *p* = 0.175 (one-sided, uncorrected), *CI* = 0.496–0.511); train on high vividness: *t*(32) = 2.58, *p* < 0.0001 (one-sided, uncorrected), *CI* = 0.503–0.523; train on low vividness: *t*(32) = 0.4, *p* = 0.77 (one-sided, uncorrected), *CI* = 0.488-0.508. ^*^p < 0.05; ^**^p < 0.005; ^***^p < 0.0005. Source data are provided as a Source Data file.

In line with our hypothesis, imagery vividness and perceptual visibility both correlated with activation in the same brain areas, despite the different nature of the task: the pre-SMA, anterior insula and right dlPFC (Fig. 3A). This coding appeared stronger during perception, possibly because high-visibility perception is generally subjectively stronger than even the most vivid imagery^19^. Furthermore, using cross-decoding multivariate pattern analysis (after mean-centring the activation within imagery and within perception to account for mean differences in strength), we found that the manner in which high versus low visibility and vividness was encoded in each area was also similar, leading to significant cross-decoding within each ROI (pre-SMA: *M* = 0.53, *SD* = 0.06, *t*(34) = 2.96, *p* = 0.0003, *d* = 0.5, *CI* = 0.51–0.55; anterior insula: *M* = 0.52, *SD* = 0.04, *t*(34) = 2.96, *p* = 0.0008, *d* = 0.5, *CI* = 0.51– 0.54; right dlPFC: *M* = 0.52, *SD* = 0.05, *t*(34) = 2.37, *p* = 0.0097, *d* = 0.4, *CI* = 0.502–0.53). Note that this analysis did not investigate the neural correlates of the *content* of imagery and perception, but rather their strength or vividness, which might explain the absence of visual cortex involvement. The notion of higher-order, abstract (content-free) neural codes for perceptual strength has attracted much recent debate in both philosophy and psychology^41^ but direct empirical evidence for such magnitude representations has been lacking. Our results suggest that the strength of visual experience is encoded in similar activity patterns, regardless of whether it reflects imagery or perception. The functional nature of such signals remains to be fully understood. For instance, one possibility is that these areas might reflect changes in (a model of) visuospatial attentional strength which is in turn used to infer both perceptual visibility^42,43^ and imagery vividness.

We did not find any direct effects of imagery vividness or perceptual visibility on univariate activation in sensory areas, despite previous studies suggesting a key role for the early visual cortex (EVC) in determining the vividness of visual experience^44^. Importantly, however, vividness might not be related to overall activation levels, but rather to how precisely stimulus information is encoded in activation patterns. For instance, previous studies have found that imagery vividness is related to the extent of representational overlap between imagery and perception in EVC – i.e. the more vivid the imagery, the more perception-like the stimulus representation in EVC^45–47^. Our source mixing model nuances this hypothesis, and predicts that not only should high-vividness imagery be more perception-like than low-vividness imagery, but also that low-visibility perception should be more imagery-like than high-visibility perception. To further investigate the influence of vividness/visibility on sensory activity, we ran a region of interest (ROI) analysis on the activation in EVC (Fig. 3B). In line with our whole-brain analysis, we did not find a direct effect of either imagery vividness (*F*(26,3) = 1.76, *p* = 0.18; Fig. 3B, top left plot) or perceptual visibility (*F*(26,3) = 0.35, *p* = 0.78; Fig. 3B, top middle plot), nor any above-chance cross-decoding between vividness and visibility (*M* = 0.51, *SD* = 0.03, *t*(34) = 1.18, *p* = 0.13, CI = 0.5–0.52; Fig. 3B, top right plot) in EVC activation. However, we did find that representations of stimulus content were modulated by vividness and visibility. In line with our hypothesis, we could decode stimulus content on perception trials significantly better from high vividness imagery trials (*M* = 0.513, *SD* = 0.029) than from low vividness imagery trials (*M* = 0.498, *SD* = 0.029, *t*(32) = 2.50, *p* = 0.017, *d* = 0.5, *CI* = 0.006–0.02), indicating that perception is more akin to highly vivid imagery. We were also able to significantly decode the identity of imagined stimuli from a classifier trained on low visibility (*M* = 0.504, *SD* = 0.03, *p* = 0.025, *d* = 0.13, *CI* = 0–0.014), but not from high visibility perception trials (*M* = 0.503, *SD* = 0.022, *p* = 0.175), suggesting that imagery is akin to low visibility perception (although this difference was itself not significant *t*(32)  = −0.15, *p* = 0.88).

### Determining whether something is real

Our findings suggest that imagery and perception are subjectively intermixed. How, then, do we ever determine whether something is real? According to our model, this is achieved simply by evaluating whether the total strength of a signal exceeds a reality threshold (Fig. 1B; right–dashed black line), based on the assumption that imagery is generally weaker, or less vivid, than perception^19,20^. Such a model predicts that reality monitoring should be worse in people with more vivid imagery. In line with this idea, we found that the frequency of source confusions – mistaking reality for imagination or imagination for reality – was associated with generally higher imagery vividness across subjects (Supplementary Fig. 4). Specifically, in experiment 1, during the congruent condition, average vividness ratings on the trials preceding the critical trial were higher for participants who erroneously judged real stimuli to be imagined (*M* = 2.48, *SD* = 1.01) compared to participants who correctly judged those stimuli to be real (*M* = 2.11, *SD* = 1.09; *t*(144) = 2.04, *p* = 0.043, *d* = 0.35, CI = 0.02–0.72), but not during the incongruent condition (*t*(124) = 1.19, *p* = 0.234). Furthermore, in the no-input Experiment 2, mean pre-critical-trial vividness was higher for participants who erroneously thought a real stimulus had been presented (*M* = 2.78, *SD* = 0.92) compared to participants who accurately judged their sensory experience to be imagined (*M* = 2.24, *SD* = 0.96; *t*(337) = 3.38, *p* = 0.0008, *d* = 0.57, CI = 0.23–0.85).

## Discussion

In this study we investigated how imagined and perceived signals interact to determine reality judgements. By combining large-scale single-trial psychophysics, computational modelling and neuroimaging, we find evidence in support of a theoretical model in which reality and imagination are intermixed to determine a unified sensory experience. This model runs counter to accounts in which imagery and perception are separable, and to earlier findings of the Perky effect which imply imagery suppresses perception of reality. When deciding whether an experience reflects external reality or internal imagination, our model compares the strength of this experience to a reality threshold. But if reality and imagination are subjectively intermixed by default, why do we not confuse them more often in daily life? We suggest that such confusions are rare simply because imagery is typically less vivid than veridical perception, rarely crossing the reality threshold. However, these results also suggest that if imagery does become vivid or strong enough, it will be indistinguishable from perception.

Our findings are inconsistent with a Perky effect, in which people tend to downweigh or discard incoming sensory information when imagining. More broadly, our results challenge a proposal that the intention or volition associated with imagery is used to classify an experience as imagined rather than real. According to a range of theoretical frameworks, sensory signals that can be predicted from top-down intentions are tagged as self-triggered and external input congruent with these predictions is suppressed^11,13,15,48–55^. One possible explanation for the discrepancy between these proposals and our results is that the sensory signals caused by our own actions – such as shifts in visual input caused by eye movements, proprioceptive signals caused by arm movements and auditory signals caused by speech production – tend to be stronger than those caused by visual imagery, and therefore may be more in need of suppression. Indeed, the sensory signals produced by self-action are often comparable in strength to those same signals caused by external sources – i.e. the world actually shifting, your arm being moved or someone else’s speech being heard. In contrast, due to the absence of excitatory, bottom-up input, the sensory signals caused by visual imagery are generally much weaker than those caused by external visual signals^20,21^. Accordingly, while there is a clear need to attenuate self-triggered signals during overt action, such a mechanism might not be necessary for attenuating self-triggered signals during covert imagery. In line with this idea, there is evidence that auditory and visual hallucinations rely on (partly) distinct mechanisms^48,55^. Future studies investigating the influence of visual imagery on simultaneously congruent perception in different scenarios as well as the neural consequences of congruent imagery on sensory processing of external inputs are necessary to test this hypothesis.

If congruent sensory input is not suppressed during mental imagery, why did Perky then observe such a clear suppressive effect of imagery in her study? One possibility is that Perky’s finding was a false positive, caused by the low statistical power of her sample. Another, more interesting possibility is that the reality threshold is likely to be dynamic and influenced by several factors. Both in the lab and in the world, the current context provides information about the likelihood of an external stimulus being presented. In Perky’s experiment, during which stimuli were presented using a hidden ‘magic lantern’, and in an era when video presentations were very uncommon^56^, participants may have been more likely to believe that their sensory experience was a product of their imagination – because what else could have caused it? This might also partly explain the steady decline of the Perky effect over the last century when technological advances made visual presentation of stimuli more and more common, leading to a decrease in reality thresholds (Supplementary Fig. 1). An exciting avenue for future research is to further characterize the contextual and cognitive factors that influence the reality threshold and how this is implemented in the brain. A final possibility is that there is a difference between knowing something is real and experiencing it as real^21^. Perhaps participants in Perky’s experiment cognitively attributed their sensory experience to imagery, because they had no alternative explanation, but due to the enhanced strength of their experience caused by the external input, they still experienced it as real. This would be in line with participants saying things like “if they hadn’t known they were imagining, they would have thought it real” (Perky, 1910; p. 433).

Our source mixing model for perceptual reality monitoring has intriguing clinical implications. A model in which sensory strength biases source judgements towards reality is in line with a proposal that hallucinations are caused in part by hyperactivation of sensory areas^57–59^. Within our source mixing model, such hallucinations would arise when internally triggered activity is so strong that it crosses a reality threshold. Reality monitoring errors could alternatively also be caused by problems with setting a reality threshold. One factor that might influence this calibration is individual differences in imagery vividness, with more vivid imagery rendering it more difficult to choose a setting of the reality threshold that cleanly separates reality from imagination. This hypothesis is in line with our findings that people who reported higher imagery vividness in general (on the trials preceding the critical trial) were more likely to exhibit failures of perceptual reality monitoring. Furthermore, more vivid imagery has been associated with an increased probability of experiencing hallucinations in both clinical^60,61^ as well as non-clinical populations^62^ (however, see^63^). Future studies could investigate whether the reality monitoring errors found in the current study are indeed associated with a higher probability of experiencing hallucinations in both clinical and non-clinical samples, as would be predicted by our source mixing model.

The exact mechanism through which imagery and perception are intermixed in the brain is an interesting question for future research. One possibility is that imagery amplifies perceptual signals in a similar way as top-down, feature-based attention. However, two recent studies suggest that imagery and feature-based attention might rely on different mechanisms. First, people with aphantasia – an inability to form mental images – perform just as well as controls on a feature-based attention task^64^. Second, while feature-based attention provides a signal-dependent boost in sensory input, leading to an increase in perceptual sensitivity, imagery appears to add sensory evidence irrespective of input strength^65^. Moreover, an explanation of intermixing in terms of top-down attention only accounts for imagery’s influence on perception but does not explain why perception in turn should also increase imagery vividness. Another possibility is that the intermixed sensory representation is read out by higher-order brain areas to determine both its strength and source. This idea would neatly fit with our neuroimaging results showing that frontal areas code both perceptual and imagery vividness while sensory areas encode stimulus content, and would be in line with both higher-order theories of consciousness and recent models of the mechanisms supporting perceptual reality monitoring^21,66–68^.

Taken together, our results reveal a subjective intermixing of imagery and perception, leading to widespread perceptual reality monitoring failure in large general population samples. The success of a signal detection model in capturing these data patterns indicates that reality monitoring may be implemented simply by comparing sensory signals against a reality threshold. Such a model is parsimonious and powerful, but also has profound implications. In particular, a consequence of this account is that it predicts when virtual or imagined sensory signals are strong or detailed enough, they become indistinguishable from reality. While currently the most common driver of top-down signals is imagination, in near-future scenarios in which brain stimulation and/or virtual reality technology is used to drive sensory signals we might be less able to tell apart reality from unreality than we would like to believe. Our model provides a framework within which to investigate these issues, and offers a route towards ensuring our reality thresholds remain well-calibrated in the face of technological advances.

## Methods

The experimental design and initial analyses were preregistered within the Open Science Framework (OSF; https://osf.io/rdqvm/?view_only=ec5e3e7afd78409cb9b4419c1ae41902) unless otherwise stated. The computational modelling and neuroimaging analyses were not included in the pre-registration document.

### Computational models

In all models we assume that the observer’s percept $P$ and imagery vividness $I$ are both a function of two random variables, a perceptual sample $X$ and a vividness sample $V$.

1 $$P=f\left(X,V\right)$$

2 $$I=f\left(X,V\right)$$

In keeping with standard signal detection theory (SDT) approaches, the perceptual sample is drawn from a bivariate Gaussian with mean ${\mu }_{X}$ and covariance ${\Sigma }_{X}$:

3 $$X \sim N({\mu }_{X},\,{\Sigma }_{X})$$

where ${\mu }_{X}=[1\,0]$ for left-tilted stimuli, $[0\,1]$ for right-tilted stimuli, and $[0\,0]$ for stimulus absence.

The vividness sample is drawn from a bivariate Gaussian with mean ${\mu }_{V}$ and covariance ${\Sigma }_{V}$:

4 $$V \sim N({\mu }_{V},\,{\Sigma }_{V})$$

where ${\mu }_{V}=\left[{V}_{S}\,0\right]$ or $\left[0\, {V}_{S}\right]$, depending on the stimulus (left-tilt or right-tilt) the observer has been asked to imagine on this trial.

${V}_{S}$ reflects a subject’s average imagery vividness, which is itself drawn from a normal distribution:

5 $$\,{V}_{S} \sim N(2.5,\,1)$$

Due to the symmetry of left- and right-tilted stimuli, when simulating the model, we assumed imagery was always for left-tilted stimuli (${\mu }_{V}=\left[{V}_{S}\,0\right]$) and varied whether ${\mu }_{X}$ was congruent or incongruent (or absent) with respect to this imagery. ${\Sigma }_{X}$ and ${\Sigma }_{V}$ were both set to the identity matrix.

Depending on the model variant, *P* and *I* are then formed via different decision rules.

*Model 1 – source separation*

6 $$P=X$$

7 $$I=V$$

*Model 2 – Perky effect*

8 $$\,P=\alpha X-V$$

9 $$\,I=V$$

*Model 3 – source mixing*

10 $$P=I=V+\alpha X$$

*Model 3b – two-parameter source mixing*

11 $$P=X+\alpha V$$

12 $$I=V+\beta X$$

Note that Model 3 is a special case of Model 3b when $\alpha$ =1 and $\beta$ = 1.

In all models the integrated percept $P$ is then compared to a reality threshold *T* to determine reality monitoring decisions (real, imagined), which we assumed to be fixed and equal to mean vividness across subjects (2.5). If $P \, > \,{T}$, the trial is classified as real, and imagined otherwise.

### Large-scale online psychophysics

#### Participants

400 participants were recruited for experiment 1 (100 per condition: imagery (left vs. right tilt) x perception (left vs. right tilt)) using Prolific (www.prolific.co) and completed the study online. Data were collected on a private institutional server managed by the JATOS tool^69^. Informed consent was obtained from each participant included in the study. The study took approximately 10 min to complete and participants were paid £1.25 for their contribution, equivalent to an hourly rate of £7.50. All procedures were approved by the University College London Research Ethics Committee. Due to the single-critical-trial design of our experiment, we were unable to determine individual threshold contrast for each participant a priori, and instead we estimated participants’ discrimination sensitivity at the presented contrast after the main task (see below for more details). This meant that we had to exclude a high number of participants who would have been unable to detect the stimuli in the main task due to low sensitivity. 4 participants were removed due to technical issues, 4 because they participated in multiple conditions, 73 because of having discrimination performance below 55%, 11 because they indicated in the debrief questions not to have imagined the stimuli as instructed and 36 because they indicated the presence of the incorrect stimulus (see below). The final sample consisted of 272 participants (mean age 27.5, *SD* 9.9), 146 in the congruent condition and 126 in the incongruent condition. For experiment 2, we collected participants as above until we obtained 40 usable participants who reported the presence of a grating despite none being presented, to provide a sufficient between-subject false alarm rate for analysis. This led to the collection of 461 participants. 4 of these were removed because they participated multiple times, 87 because of having discrimination performance below 55%, 12 because they indicated in the debrief questions not to have imagined the stimuli as instructed and 23 because they indicated the presence of the incorrect stimulus. The final sample for experiment 2 contained 339 participants (mean age 27.5, *SD* 10.1)

#### Experimental procedures and design

Prior to the start of the main task, participants filled out the Vividness of Visual Imagery Questionnaire (VVIQ^70,71^). The VVIQ measures people’s general imagery vividness and also serves the purpose of clearly explaining the concept of mental imagery and the imagery vividness scale. During the main task, participants were instructed to imagine a left or right tilted grating as vividly as possible while looking at dynamic noise (Fig. 1B; main text). After each trial, participants were asked to rate their imagery vividness on a scale from 1 (not vivid at all) to 5 (as vivid as real seeing). Participants performed 10 of these imagery trials and on the 10th trial either the same grating (congruent) or a grating orthogonal to the imagined grating (incongruent) gradually ramped up to about threshold. After rating the vividness of the 10th trial, participants were asked: “On the last trial, was there a grating presented on the screen?” The response options were: “No, there was only noise on the screen, any grating I saw was my imagination [No]; Yes, there was actually a left-tilted grating on the screen [Left tilted]; Yes, there was actually a right-tilted grating on the screen [Right tilted].” After this, they were asked to rate their confidence in this decision on a five-point scale.

Since there was only one detection trial per participant we were not able to determine threshold contrast values on an individual basis. Instead, we selected the contrast value that was associated with an average detection performance of ~80% in a previous sample. We first ran the study with a visibility level of 4% grating in noise, which was associated with detection performance around 70% in a previous sample. However, this resulted in only 12% ‘real’ responses during the critical trial and of these, only 57% were for the correct grating (chance is 50%), suggesting that the contrast was too low for participants to be able to perceive the presented grating during the critical trial. Therefore, in the experiments reported here, the visibility level was increased to 7%. With this higher visibility level the proportion of presence responses during the critical imagary trial was still relatively low (44% on average over conditions), but 72% of those were for the correct grating, indicating that participants were able to sometimes perceive the (correct) grating.

Differences in viewing distance and monitor brightness between participants likely resulted in considerable variation in grating visibility between participants. Therefore, to ensure that all included participants could detect the stimuli at this contrast level, after the main experiment participants performed a standard forced-choice discrimination task on the left and right-tilted gratings presented at the same contrast in the same dynamic noise schema for 40 trials. We chose a discrimination task instead of a detection task to avoid measurement of perceptual sensitivity being influenced by differences in detection criterion. Furthermore, discrimination and detection performance can be mathematically related under signal detection theory (SDT), allowing us to use discrimination sensitivity to infer detection sensitivity^72^. Interesting, despite the low hit rate during the reality judgement, detection sensitivity as estimated using the discrimination task with the same visibility level but without imagery was high and significantly above chance (d’ *M* = 2.1, *SD* = 0.73). Finally, there was a significant negative correlation between averaged pre-critical trial imagery vividness and discrimination sensitivity in both experiment 1 (*r* = −0.2, *p* = 0.0007) and experiment 2 (*r* = −0.18, *p* = 0.0007).

Finally, it is possible that participants were unsure what gratings at threshold were supposed to look like on the critical trial, limiting our ability to characterise reality monitoring failures. Therefore, after participants performed the discrimination task (and had plenty of experience with the stimuli involved) they were asked again whether they saw a grating on the critical imagery trial and asked to again rate their confidence in this response. Of the included participants 150/272 (55%) changed their judgement after the discrimination task in experiment 1 and 204/339 (60%) in experiment 2. These changes of mind consisted mostly of ‘imagined’ responses being changed to ‘real’ responses after the discrimination task (75% of the changes of mind in experiment 1 and 89% in experiment 2), which is consistent with the discrimination task revealing to participants both a) that real near-threshold gratings are commonplace in our task environment and b) that such gratings are often difficult to see. As we already found clear results with the immediate reality monitoring judgement, the data of this second judgement were not analysed further. Importantly, the qualitative pattern of results (condition effects on proportion real judgments and vividness reports) was similar when looking at the second response, but given the delay, we considered the immediate response to be a more reliable measure of source confusion.

#### Data analysis and statistics

Analyses were performed in MATLAB R2018b, JASP 0.14.1.0^73^ and SPSS 25^74^. To investigate whether external input was mistaken for imagery, we tested whether imagining a congruent stimulus was associated with a different (i.e. two-sided) probability of reporting real stimulus presence compared to imagining an incongruent stimulus. To this end, we used binary logistic regression with response (“real” versus “imagined”) as the dependent variable and condition (congruent vs. incongruent) as the independent variable. “Imagined” responses were coded as zeros and correct presence (“real”) responses as ones. We removed participants who indicated that they perceived a grating orthogonal to the presented grating because these responses are hard to interpret (they might have reflected hallucinations in the context of incongruent imagery, but it is unclear what they reflected during congruent imagery) and they are not essential for answering our main question.

We also measured participants’ detection sensitivity and imagery vividness. Detection sensitivity was calculated based on performance in the discrimination task as $\frac{{d}_{{discrimination}}^{{\prime} }}{\sqrt{2}}$. We decided to use the mean vividness rating on the non-critical trials as a measure of participants’ imagery vividness instead of the VVIQ because previous studies found that within-task measures were more predictive of task effects^35,45,75–78^. Consistent with the possibility of such dissociations, we found no effect of vividness as measured by the VVIQ on reality judgement responses (*b* = 0.317, *p* = 0.233), despite effects on the within-task vividness ratings (see main text). Similarly, while there was a robust correlation between the two vividness measures both in experiment 1 (*R*^2^ = 0.073, *p* = 0.000006) and experiment 2 (*R*^2^ = 0.07, *p* = 0.00001), a large proportion of the variation in vividness measured by the online ratings was not captured by the VVIQ. We therefore decided to focus on within-task vividness ratings as our primary individual difference measure of mental imagery. Nonetheless, despite the absence of any task effects, the VVIQ was still useful as a validated way to explain the concept of mental imagery to our participants.

We expected the probability of reporting stimulus presence to be positively correlated with detection sensitivity and negatively with imagery vividness. Furthermore, we hypothesized that these factors might interact with congruency such that source confusion, measured as the difference between congruent and incongruent imagery, would be largest for participants with low detection sensitivity and high imagery vividness. We tested this by adding detection sensitivity and imagery vividness as well as their interactions (both with each other and with congruency) as predictors to the logistic regression model.

We used backwards selection to determine which factors best predicted the presence responses. The model with the best fit, measured as the lowest Bayesian Information Criterion, included only the main effects of condition (congruent vs. incongruent), detection d’ and imagery vividness as predictors (*X*^2^(1) = 28.442, *p* < 0.001). This model explained 13.8% (*Nagelkerke R*^2^) of the variance in reality judgements and correctly classified 70.6% of cases. The main effects of condition are reported in detail in the main text. Detection sensitivity, as measured by the discrimination task, also significantly predicted the likelihood of reporting presence of a real stimulus (*β* = 0.841, *SE* = 0.233; *p* < 0.001).

Finally, we considered that if participants indeed confused external signals as imagery, this might also manifest in subjects’ confidence reports during the critical reality judgement trial. Specifically, stimulus-presence judgements might be held with less confidence in the congruent compared to the incongruent conditions, due to the source confusion inherent to this condition. We tested this using multinomial ordinal regression with confidence as dependent variable and condition as independent variable. There was no significant effect of either condition (*t*(1) = 0.020, *p* = 0.984), response (*t*(1) = 0.753, *p* = 0.451) or their interaction (*t*(1) = 0.178, *p* = 0.859) on confidence.

### Neuroimaging data and analyses

To investigate the relationship between neural coding of imagery vividness and perceptual visibility, we re-analysed previously acquired fMRI data. More information about task design, participants and pre-processing of the fMRI data can be found in ref. ^40^.

#### Participants and experimental design

fMRI data from 35 participants were analysed (mean age, 25.9 years; SD  =  5.9). The imagery task was a retro-cue task in which two stimuli were presented after each other for 500 ms each followed by a retro-cue indicating whether the first or second stimulus was to be imagined. The participant then imagined this retro-cued stimulus for 4s^79^. The perception task consisted of a backwards masking design in which the stimulus was presented for one frame (17 ms) followed by an ISI of 66 ms and a mask of 400 ms. After each imagery or perception trial, participants decided whether the perceived or imagined stimulus was animate or inanimate. Stimuli were 4 full-colour objects/animals: a watering can, a football, a fish and a rooster. After the discrimination decision, participants rated their imagery vividness/perceptual visibility on a scale from 1 to 4. Response mappings (whether 1 indicated high or low visibility) were randomized over trials so that visibility modulations cannot reflect motor-related activity.

#### Data analysis and statistics

Analyses were performed in MATLAB R2018b. First, trial specific beta maps were obtained using SPM12 within a GLM with one regressor per trial plus nuisance regressors (e.g. movement, instruction screens, button presses). Next, we determined which brain areas were modulated by imagery vividness and perceptual visibility by running a GLM on the trial-specific beta values with a regressor for the main effects of perception and imagery as well as the parametric modulation of vividness and visibility respectively. Significance was determined by comparing the betas for vividness and visibility at the group level against 0 with a one sample *t*-test and then correcting the *p*-value for multiple comparisons using FDR correction at a *q*-value of 0.01.

We used the AICHA atlas^80^ to determine regions of interest (ROIs) for cross-decoding of vividness and visibility, based on which brain areas were revealed as being univariately modulated by vividness and visibility. We identified three ROIs: pre-SMA (containing ‘G_Frontal_Sup_Medial-3107’,’G_Supp_Motor_Area-1112’ and ‘S_Cingulate-1115’), anterior insula (containing ‘G_Insulate_Anterior-376’ and ‘G_Insulate_Anterior-477’) and dorsolateral prefrontal cortex (containing ‘S_Inf_Frontal-116’ and ‘S_Inf_Frontal-217’). Cross-decoding was performed by training a classifier on low (ratings 1 and 2) versus high (ratings 3 and 4) imagery vividness and then testing it on high versus low perceptual visibility and vice versa. We then averaged over decoding directions to calculate the cross-decoding accuracy^81^. Decoding was done using a linear discriminant analysis, as in ref. ^82^. Prior to decoding, data were down-sampled so that there were an equal number of trials in the low and high class, and mean-centred so that overall amplitude differences between imagery and perception were removed and only relative differences between high and low vividness/visibility remained. Participants with fewer than 10 trials per class were removed from this analysis (*N* = 4).

To explore to what extent activation in visual cortex was modulated by vividness, we performed an ROI analysis on the early visual cortex (EVC). The EVC ROI was created by combining all early visual areas (V1-V4) from the Kastner atlas^83^. Stimulus identity was decoded in a pairwise fashion and subsequently averaged over all pairs to create a stimulus decoding summary statistic for each classification type. Classifiers were trained on low (ratings 1 and 2) versus high (ratings 3 and 4) imagery vividness/perceptual visibility trials and tested on all perception/imagery trials to investigate generalization of decoding. Participants with fewer than 2 trials per stimulus class per visibility/vividness level were removed from the analysis (*N* = 2).

Determining whether decoding accuracy was significantly above-chance was done by comparing mean group decoding accuracy with a null distribution created by permuting the class labels prior to decoding 25 times per participant and then using bootstrapping to create a group null-distribution^84^. Comparisons between decoding accuracies were done using *t*-tests.

### Reporting summary

Further information on research design is available in the Nature Portfolio Reporting Summary linked to this article.

## Supplementary information

Supplementary info

Reporting Summary

## Data Availability

The psychophysical data generated in this study have been deposited in GitHub [https://github.com/NadineDijkstra/IMAREAL]. The raw MRI data have been deposited in the Donders Repository [10.34973/j9yn-q419]. The pre-processed data used for figure generation are available in the accompanying Source Data File. The atlases used in this study are openly available. The AICHA atlas is part of the SPM12 toolbox and the Kastner atlas can be accessed online [https://pubmed.ncbi.nlm.nih.gov/25452571/]. Source data are provided with this paper.

## Source data

Source Data

## Code availability

All experimental and analysis code is available at [10.5281/zenodo.7646917].
