# Supplementary material for: Subjective signal strength distinguishes reality from imagination
Source: Nat Commun. 2023 Mar 23;14:1627. doi: 10.1038/s41467-023-37322-1 (PMC10036541; doi:10.1038/s41467-023-37322-1)
Supplement: Supplementary file 1 — Supplementary info [file 41467_2023_37322_MOESM1_ESM.pdf]

Supplementary Material

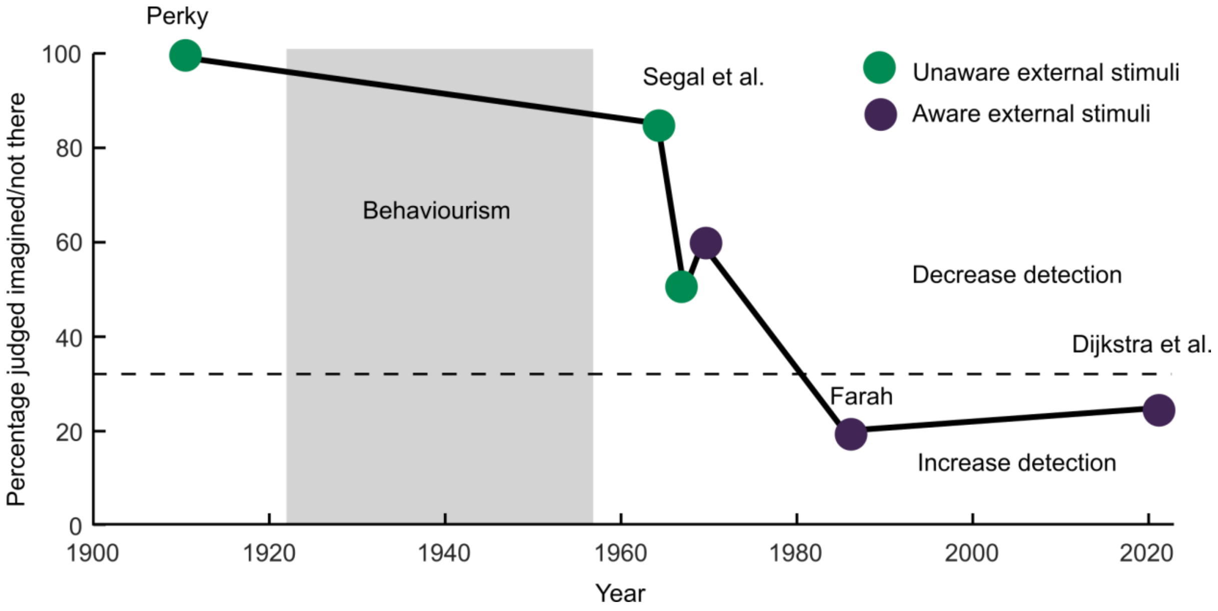

Supplementary Figure 1. Empirical history of perceptual reality monitoring. Empirical investigation of reality monitoring was initiated by the seminal work of Mary Cheves Perky in 1910, using a magic lantern to project vaguely coloured congruent shapes while participants imagined various objects[56]. She showed that in 100% of the cases, projected stimuli were mistaken to be the result of participant's imagination. After a gap in imagination research (possibly due to the rise of behaviourism), Perky's experiments were revisited by Sydney Segal in the 60s[86–88], by Martha Farah in the 80s[34] and recently also by our group[13]. Several other important studies on the interaction between imagination and perception were done in this time period but in many studies, the imagined and perceived stimuli were not the same or were not simultaneously presented, which means that effects are unlikely to have reflected source confusion and can more easily be ascribed to other cognitive factors such as attention, see e.g.[31,89]. Over time, the percentage of cases attributed to imagination dropped, leading to a decrease and eventually even a reversal of the Perky effect in more recent studies. One possible reason for this drop is that it became the norm to test a higher number of trials in order to obtain statistically robust effects. However, within these multi-trial experiments, as soon as participants notice that external stimuli might be presented, source attribution on future trials becomes naturally biased towards reality. This leads to a conundrum of how to obtain high sufficient statistical power while at the same time avoiding biasing participants' reality judgements, which we addressed in the current study by using large-scale online psychophysics.

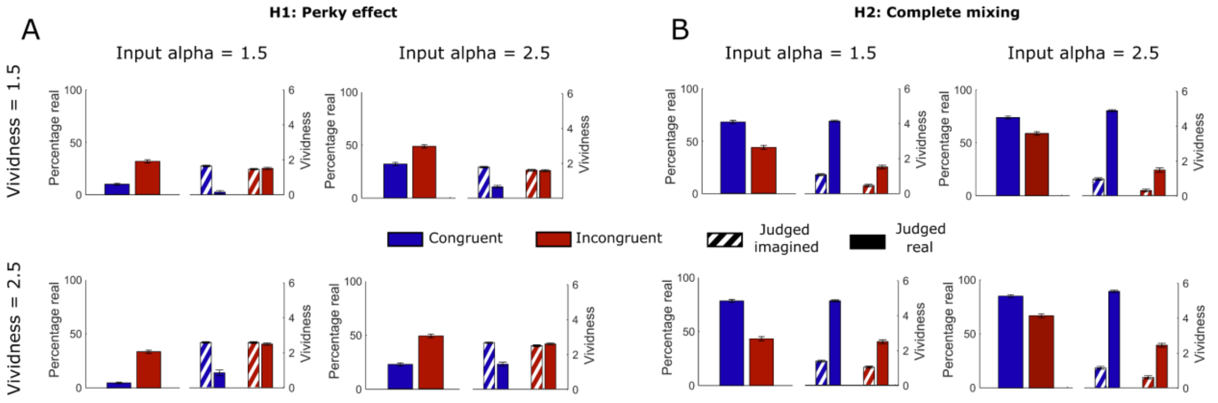

Supplementary Figure 2. Influence of task engagement on model predictions. Low task engagement could lead to a reduction in external attention, modelled here as a change in the scaling of the input (alpha) or a reduction in the imagery signal, modelled here as a change in the group vividness. **a**. Effects under the Perky model. **b**. Effects under the complete source mixing model. Changes in the input and imagery strength lead to changes in the average proportion of presence responses and reported vividness, but not to the qualitative pattern of differences between the conditions predicted by the different models. For both **a.** and **b.** data are presented as mean values +/- SEM over simulation samples. From top left to bottom right, n = 1897, 1917, 1838, 1831, 1761, 1634, 1838, 1831 independent simulation samples.

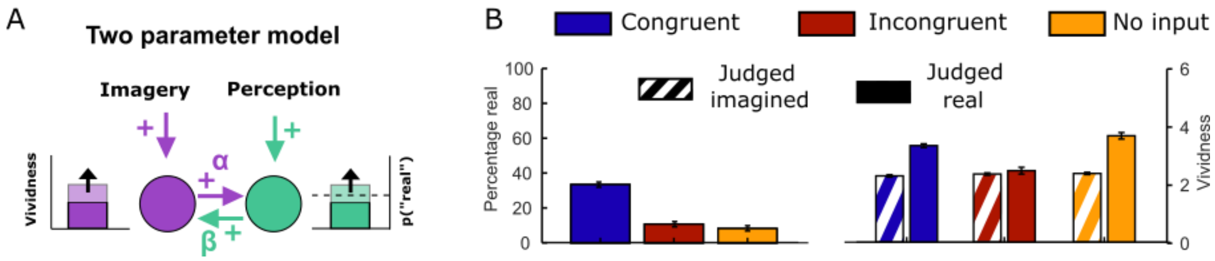

Supplementary Figure 3. Alternative two-parameter mixture model. a. Model architecture: P = X + α*V and I = V + β*X. α controls the strength of imagery's influence on perception and β controls the strength of perception's influence on imagery. **b.** Simulated results for α = 0.4 and β = 0.1. Predicted patterns are qualitatively similar to the source mixing model, cf. Fig. 2 main text. Data are presented as mean values +/- SEM over simulation samples; n = 2915 independent simulation samples.

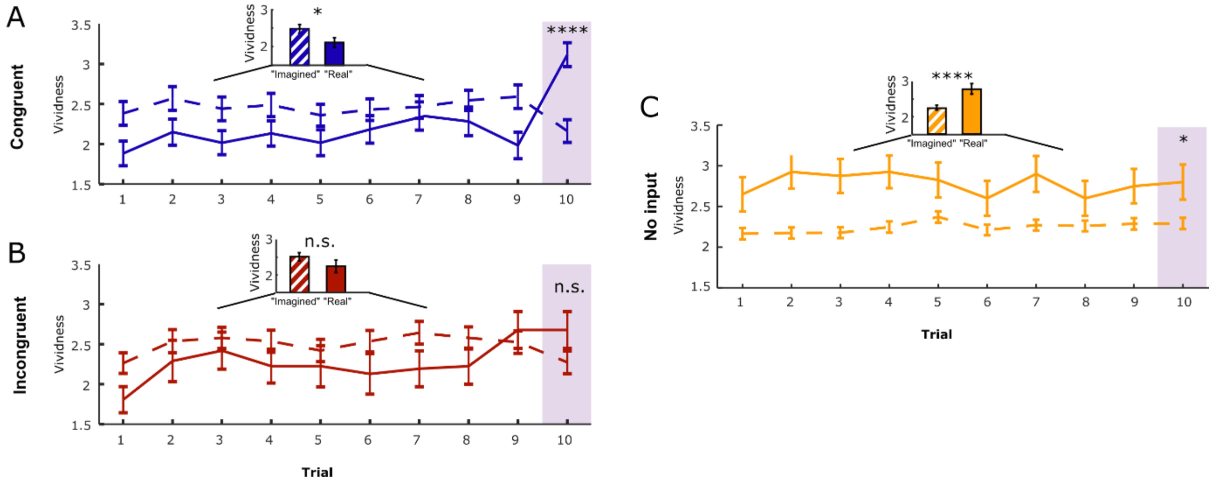

Supplementary Figure 4. Vividness ratings over the course of the experiment. Dashed lines indicate trials which were later judged to be imagined on the critical trial, solid lines indicate trials that were later judged to be real. Bar graphs indicate vividness averaged over the first 9, non-critical trials. Data are presented as mean values +/- SEM over participants. **a.** Congruent condition, pre-critical-trial: $t(144) = -2.04$, $p = 0.043$ (uncorrected), CI = -0.717—0.012; critical-trial: $\beta(1) = 0.40$ (*CI* = 0.22 – 0.58) $p = 0.000013$ (uncorrected). n = 146 independent participants. **b.** Incongruent condition, pre-critical-trial: $t(124) = -1.19$, $p = 0.235$ (uncorrected), CI = -0.718-0.178; critical-trial: $\beta(1) = 0.19$ (*CI* = -0.03 – 0.40), $p = 0.092$ (uncorrected). n = 126 independent participants. **c.** Experiment 2, no input: pre-critical-trial: $t(337) = 3.38$, $p = 0.0008$ (uncorrected), CI = 0.23-0.86; critical-trial: $\beta(1) = 0.22$, $p = 0.013$ (uncorrected), *CI* = 0.05 – 0.40. * $p < 0.05$; ** $p < 0.005$; *** $p < 0.0005$; **** $p < 0.00005$. n = 339 independent participants.
