# Supplementary material for: Subjective signal strength distinguishes reality from imagination
Source: Nat Commun. 2023 Mar 23;14:1627. doi: 10.1038/s41467-023-37322-1 (PMC10036541; doi:10.1038/s41467-023-37322-1)
Supplement: Supplementary file 2 — Reporting Summary [file 41467_2023_37322_MOESM2_ESM.pdf]

nature portfolio

Corresponding author(s): Nadine Dijkstra

Last updated by author(s): Feb 16, 2023

Reporting Summary

Nature Portfolio wishes to improve the reproducibility of the work that we publish. This form provides structure for consistency and transparency in reporting. For further information on Nature Portfolio policies, see our Editorial Policies and the Editorial Policy Checklist.

Statistics

For all statistical analyses, confirm that the following items are present in the figure legend, table legend, main text, or Methods section.

n/a	Confirmed	
☐	☒	The exact sample size (*n*) for each experimental group/condition, given as a discrete number and unit of measurement
☐	☒	A statement on whether measurements were taken from distinct samples or whether the same sample was measured repeatedly
☐	☒	The statistical test(s) used AND whether they are one- or two-sided<br>*Only common tests should be described solely by name; describe more complex techniques in the Methods section.*
☐	☒	A description of all covariates tested
☐	☒	A description of any assumptions or corrections, such as tests of normality and adjustment for multiple comparisons
☐	☒	A full description of the statistical parameters including central tendency (e.g. means) or other basic estimates (e.g. regression coefficient) AND variation (e.g. standard deviation) or associated estimates of uncertainty (e.g. confidence intervals)
☐	☒	For null hypothesis testing, the test statistic (e.g. *F*, *t*, *r*) with confidence intervals, effect sizes, degrees of freedom and *P* value noted<br>*Give P values as exact values whenever suitable.*
☒	☐	For Bayesian analysis, information on the choice of priors and Markov chain Monte Carlo settings
☒	☐	For hierarchical and complex designs, identification of the appropriate level for tests and full reporting of outcomes
☐	☒	Estimates of effect sizes (e.g. Cohen's *d*, Pearson's *r*), indicating how they were calculated

Our web collection on statistics for biologists contains articles on many of the points above.

Software and code

Policy information about availability of computer code

Data collection	Data collected was done online via Prolific (https://app.prolific.co/) using JsPsych and JATOS - all code can be found at https://github.com/IMAREAL
Data analysis	Data were analyzed using MATLAB R2018b, with SPM12 and custom code, and JASP 0.14.1.0 and SPSS 25 for statistical inference. All analysis code can be found at https://doi.org/10.5281/zenodo.7646917

For manuscripts utilizing custom algorithms or software that are central to the research but not yet described in published literature, software must be made available to editors and reviewers. We strongly encourage code deposition in a community repository (e.g. GitHub). See the Nature Portfolio guidelines for submitting code & software for further information.

Data

Policy information about availability of data

All manuscripts must include a data availability statement. This statement should provide the following information, where applicable:

- Accession codes, unique identifiers, or web links for publicly available datasets
- A description of any restrictions on data availability
- For clinical datasets or third party data, please ensure that the statement adheres to our policy

The psychophysical data generated in this study have been deposited in GitHub [https://github.com/IMAREAL]. The raw MRI data have been deposited in the

Donders Repository [https://doi.org/10.34973/j9yn-q419]. The pre-processed data used for figure generation are available in the accompanying Source Data File. The atlases used in this study are openly available. The AICHA atlas is part of the SPM12 toolbox and the Kastner atlas can be accessed online [https://pubmed.ncbi.nlm.nih.gov/25452571/].Donders Repository [https://doi.org/10.34973/j9yn-q419]. The pre-processed data used for figure generation are available in the accompanying Source Data File. The atlases used in this study are openly available. The AICHA atlas is part of the SPM12 toolbox and the Kastner atlas can be accessed online [https://pubmed.ncbi.nlm.nih.gov/25452571/].

Human research participants

Policy information about studies involving human research participants and Sex and Gender in Research.

Reporting on sex and gender	In line with the data minimization principle of the GDPR, we did not collect gender or sex information of our participants because we did not have any a priori hypotheses or research questions about this. Neither sex nor gender is likely to influence our findings.
Population characteristics	See behavioural and social sciences study design below
Recruitment	Participants were recruited online via Prolific (https://app.prolific.co/) which includes participants from most OECD countries. All Prolific participants are over 18 years old and have been recruited to Prolific via social media or word of mouth. The only selection criterion required for this study was that participants should not have previously participated in a similar study from our lab. This ensured a diverse sample in terms of age, gender, SES, ethnicity and education level.
Ethics oversight	University College London Research Ethics Committee

Note that full information on the approval of the study protocol must also be provided in the manuscript.

Field-specific reporting

Please select the one below that is the best fit for your research. If you are not sure, read the appropriate sections before making your selection.

☐ Life sciences ☒ Behavioural & social sciences ☐ Ecological, evolutionary & environmental sciences

For a reference copy of the document with all sections, see nature.com/documents/nr-reporting-summary-flat.pdf

Behavioural & social sciences study design

All studies must disclose on these points even when the disclosure is negative.

Study description	The psychophysical part of this study was a one-trial-per-participant between-subject design with one main binary outcome variable (reality judgment) and one main ordinal outcome variable (vividness rating). The functional MRI part of this study was a within-subject design with modality (imagery vs perception), vividness/visibility rating and stimulus identity as predictors and neural activity (BOLD signal) as outcome variable.
Research sample	We collected data from 400 participants for experiment 1 (final sample = 272, mean age 27.5, SD 9.9) and from 461 participants for experiment 2 (final sample = 339, mean age 27.5, SD 10.1). Data were collected online via Prolific (https://app.prolific.co/), a participant recruitment platform containing participants from diverse backgrounds world-wide. Sample sizes were decided based on a trade-off between statistical power and costs. More detailed information regarding the fMRI data-set reanalysed in this paper can be found at https://doi.org/10.1523/ENEURO.0228-21.2021
Sampling strategy	Random sampling from the Prolific platform was performed for both experiments. For experiment 1, sample size was based on a balance between costs and power: a pilot experiment revealed that the exclusion rate for this experiment was quite high (~40%) due to the inability to staircase visibility level at the individual subject level. Therefore, we aimed to collect data so that we had at least 100 usable participants in each condition, giving us enough power to detect effects. For experiment 2, we collected participants until we obtained 40 usable participants that reported the presence of a grating despite none being presented, to provide a sufficient between-subject false alarm rate for analysis.
Data collection	Data collection was carried out online via Prolific and JATOS. After participants agreed to take part in the study in Prolific, they were linked to the JATOS study page. Before starting the main experiment, participants had to read and sign a consent form. The experiment then started with the Vividness of Visual Imagery Questionnaire (VVIQ). After this, participants were instructed about the main task and executed 10 trials. Finally, participants performed a visual detection task to measure sensitivity to the contrast level used in the experiment. At the end, participants had the option to provide comments about their experience. Due to the online nature of the experiment, the exact conditions (e.g. whether anyone else was present) are unknown.
Timing	Data collection for experiment 1 was performed between 16th and 20th November 2020 and data collection for experiment 2 was performed between 9th and 16th December 2020.
Data exclusions	For experiment 1, 4 participants were removed due to technical issues, 4 because they participated in multiple conditions, 73 because of having discrimination performance below 55%, 11 because they indicated in the debrief questions not to have imagined the stimuli as instructed and 36 because they indicated the presence of the incorrect stimulus (see text). For experiment 2, 4 of these

were removed because they participated multiple times, 87 because of having discrimination performance below 55%, 12 because they indicated in the debrief questions not to have imagined the stimuli as instructed and 23 because they indicated the presence of the incorrect stimulus.

Non-participation: No participants dropped out/declined participation

Randomization: All conditions were published as separate experiments online simultaneously so that participation in each condition was random.

Reporting for specific materials, systems and methods

We require information from authors about some types of materials, experimental systems and methods used in many studies. Here, indicate whether each material, system or method listed is relevant to your study. If you are not sure if a list item applies to your research, read the appropriate section before selecting a response.

Materials & experimental systems

n/a	Involved in the study
☒	☐ Antibodies
☒	☐ Eukaryotic cell lines
☒	☐ Palaeontology and archaeology
☒	☐ Animals and other organisms
☒	☐ Clinical data
☒	☐ Dual use research of concern

Methods

n/a	Involved in the study
☒	☐ ChIP-seq
☒	☐ Flow cytometry
☐	☒ MRI-based neuroimaging

Magnetic resonance imaging

Experimental design

Design type: Block (modality - imagery vs perception) and event-related (stimulus identity and vividness) design

Design specifications: Each subject executed four imagery blocks (144 trials) and four perception blocks (184 trials). Imagery trials lasted ~10s and perception trials lasted ~5s depending on reaction time of the participant. The inter-trial-interval for the perception task was between 6.3 and 8.3s and for the imagery task between 4.6 and 6.6s.

Behavioral performance measures: Button presses and response times were recorded for vividness ratings and animacy ratings for both tasks. To ensure participants performed the task correctly, a d' (discrimination sensitivity) above 0 was required. Furthermore, participants were only included in analyses on vividness/visibility if they indicated more than 1 vividness level.

Acquisition

Imaging type(s): Functional and structural scans were performed

Field strength: 3T

Sequence & imaging parameters: Multiband 6 sequence (TR, 1 s; voxel size, 2×2 × 2 mm; TE, 34ms) and 32-channel head coil. For all participants, the field of view was tilted −25° from the transverse plane, using the Siemens AutoAlign Head software, resulting in the same tilt relative to the individual participant's head position. T1-weighted structural images (MPRAGE; voxel size, 1×1 × 1 mm; TR, 2.3 s) were also acquired for each participant.

Area of acquisition: Whole brain scan

Diffusion MRI: ☐ Used ☒ Not used

Preprocessing

Preprocessing software: Data were preprocessed using SPM12.

Normalization: The scans were normalized to MNI space using DARTEL (diffeomorphic anatomical registration through exponentiated lie algebra) normalization and smoothed with a 6 mm Gaussian kernel.

Normalization template: MNI152

Noise and artifact removal: All functional imaging data were motion corrected (realignment) and coregistered to the T1 structural scan. A high-pass filter of 128 s was used to remove slow signal drift.

Volume censoring: n.a.

Statistical modeling & inference

Model type and settings	Effects of vividness and visibility were estimated using a mass univariate GLM with vividness, visibility and nuisance regressors (motion, instruction screen, button presses) at the first level. Significance at the group-level was determined using a second level t-test on the beta weights determined at the first level. Decoding accuracy was tested against chance using a group-level permutation analysis within each ROI (Sterzer et al., 2013).
Effect(s) tested	Vividness and visibility effects were tested against 0 with one-sample t-tests at the group level. Group decoding accuracy was tested against chance using permutation analyses.

Specify type of analysis: ☐ Whole brain ☐ ROI-based ☒ Both

Anatomical location(s)	For the early visual cortex (EVC) ROI, a probabilistic atlas was used (https://pubmed.ncbi.nlm.nih.gov/25452571/). For the three frontal regions, the AICHA atlas labels in which the significant voxels were located (pre-SMA, anterior insulate and inferior frontal) were selected and used for subsequent decoding analyses.
Statistic type for inference (See Eklund et al. 2016)	Voxel-wise.
Correction	For the univariate analyses, whole-brain FDR correction at a q-value of 0.01 was applied. No correction was applied for the decoding analyses which were focused on specific ROIs.

Models & analysis

n/a	Involved in the study
☒	☐ Functional and/or effective connectivity
☒	☐ Graph analysis
☐	☒ Multivariate modeling or predictive analysis

Multivariate modeling and predictive analysis	Voxels within a given ROI were used as features to predict visibility/vividness or stimulus identity using a linear discriminant analysis (LDA). Accuracy was calculated as the proportion of correctly classified trials and tested for significance using a label permutation procedure.
